# The Retentivity of Four Kinds of Shadowing Properties in Non-Autonomous Discrete Dynamical Systems

**DOI:** 10.3390/e24030397

**Published:** 2022-03-12

**Authors:** Yongxi Jiang, Tianxiu Lu, Jingmin Pi, Waseem Anwar

**Affiliations:** 1 College of Mathematics and Statistics, Sichuan University of Science and Engineering, Zigong 643000, China; jyx970817@163.com (Y.J.); pi1011225770@126.com (J.P.); 2 The Key Laboratory of Higher Education of Sichuan Province for Enterprise Informationalization and Internet of Things, Zigong 643000, China; 3 School of Mathematical Science, Sichuan Normal University, Chengdu 610068, China; waseemanwarijt@yahoo.com

**Keywords:** shadowing properties, non-autonomous discrete dynamical systems, set-valued systems, 54H20, 37B45

## Abstract

In this paper, four kinds of shadowing properties in non-autonomous discrete dynamical systems (NDDSs) are discussed. It is pointed out that if an NDDS has a F-shadowing property (resp. ergodic shadowing property, $\bar{d}$ shadowing property, $\underline{d}$ shadowing property), then the compound systems, conjugate systems, and product systems all have accordant shadowing properties. Moreover, the set-valued system $\left(K\left(X\right),{\bar{f}}_{1,\infty }\right)$ induced by the NDDS $\left(X,f_{1,\infty }\right)$ has the above four shadowing properties, implying that the NDDS $\left(X,f_{1,\infty }\right)$ has these properties.

## 1. Introduction

Non-autonomous discrete dynamical systems (NDDSs) are a generalization of autonomous discrete dynamical systems (ADDSs). NDDSs are more flexible for describing some dynamic and dynamical behaviors in the real world, and have important theoretical and applied value. The dynamical properties of NDDSs have attracted wide attention from scholars. Since 1996, the chaos of NDDSs began to be of concern. Currently, some studies about the sensitivity and transitivity in NDDSs can be found in [1,2,3,4]. For some studies of Li–Yorke chaos, distributional chaos, dense chaos, Ruelle–Takens chaos, or Kato’s chaos in NDDSs, see [5,6] and others.

The shadowing property of a dynamical system is one of the most important notions in dynamical systems. It is an important tool for studying the chaotic properties of discrete dynamical systems. From the numerical point of view, if a dynamical system has the shadowing property, then numerically obtained orbits reflect the real behavior of trajectories of the systems (see [7,8,9]). With the deepening of research, various new shadowing properties are emerging. The ergodic shadowing property was introduced by Fakhari in [10]. Then, the $\bar{d}$ shadowing property and $\underline{d}$ shadowing property were introduced by Dastjerdi [11], which are on the basis of the ergodic shadowing property. In addition, Oprocha [12] used the Furstenberg family to describe the shadowing property and gave the definition of the F-shadowing property. There exist abundant research results on these shadowing properties. In 2011, Niu [13] studied that if *f* has the average-shadowing property and the minimal points of *f* are dense in *X*, then *f* is weakly mixing and fully strongly ergodic. In 2017, Ma [14] determined that a nonuniformly expanding map *f* with the $\bar{d}$ shadowing property or $\underline{d}$ shadowing property is topologically transitive. In 2019, Parham [15] showed that every uniformly equicontinuous non-autonomous discrete-time system with the ordinary shadowing and topologically mixing properties has the ergodic shadowing property. In 2021, Vasisht and Das [16] gave an interrelation among the shadowing property, periodic shadowing property and local weak specification property of an expansive non-autonomous system. Koo [17] proved that an expansive system has the periodic shadowing property if, and only if, its induced hyperspatial system has the periodic shadowing property. Some scholars generalized the notion of the shadowing property to iterated function systems (IFS). In 2016, Nia [18] proved that every uniformly contracting IFS has the asymptotic average shadowing property. If a continuous surjective IFS, F, on a compact metric space, X, has the asymptotic average shadowing property, then F is chain transitive. In [18], the author determined that for every IFS with a shadowing property, chain mixing and topological mixing properties are equivalent. For more research on the shadowing property, see references [19,20,21,22,23] and other works in the literature.

However, most of the literature on shadowing properties is not discussed in NDDSs. In order to generalize the existing conclusions or obtain new results, this paper introduces the concepts of four kinds of shadowing properties in NDDSs. Then, the retentivity of them under the cases of compound, topological conjugate, or product are studied. Further, the relationship of the shadowing properties between non-autonomous discrete dynamical system $\left(X,f_{1,\infty }\right)$ and the induced set-valued system $\left(K\left(X\right),{\bar{f}}_{1,\infty }\right)$ is discussed. The structure of this paper is as follows. In Section 2, some basic definitions and concepts are introduced. In Section 3, the main results are established and proved.

## 2. Preliminaries

### 2.1. Non-Autonomous Discrete Dynamical Systems

In this paper, let X=[0,1], and the metric on *X* is denoted as *d*. $f_{n}:X\to X\left(n\in N\right)$ is a mapping sequence, and denoted by $f_{1,\infty }=\left(f_{1},f_{2},\cdots \right)$. This sequence defines a non-autonomous discrete dynamical system (NDDS) $\left(X,f_{1,\infty }\right)$. Under this mapping sequence, the orbit of a point x∈X is $Orb\left(x,f_{1,\infty }\right)=\left(f_{1}^{n}\left(x\right)\right)\left(n\in N\right)$, where $f_{1}^{n}=f_{n}\circ \cdots \circ f_{1}$, $f_{1}^{0}$ denotes the identity mapping. Similarly, $f_{n}^{k}=f_{n+k-1}\circ \cdots \circ f_{n+1}\circ f_{n}$.

For any m∈N, denote

$$h_{1}=f_{m}\circ \cdots \circ f_{1},h_{2}=f_{2m}\circ \cdots \circ f_{m+1},\cdots ,h_{p}=f_{pm}\circ \cdots \circ f_{(p-1)m+1},\cdots$$

$\left(X,h_{1,\infty }\right)$ is called a compound system of $\left(X,f_{1,\infty }\right)$. To make it easier to see the relationship between system $\left(X,h_{1,\infty }\right)$ and system $\left(X,f_{1,\infty }\right)$, the compound system $h_{1,\infty }$ is also denoted by $f_{1,\infty }^{\left[m\right]}$.

The product system $\left(X\times Y,f_{1,\infty }\times g_{1,\infty }\right)$ of NDDSs $\left(X,f_{1,\infty }\right)$ and $\left(Y,g_{1,\infty }\right)$ (the metric of *X* and *Y* are $d_{1}$ and $d_{2}$, respectively) is defined as $\left(X\times Y,\left(f_{1}\times g_{1},f_{2}\times g_{2},\cdots ,f_{n}\times g_{n},\cdots \right)\right)$, where

$$f_{n}\times g_{n}\left(\left(x,y\right)\right)=\left(f_{n}\left(x\right),g_{n}\left(y\right)\right)\;\;\;\;\;\left(\forall \left(x,y\right)\in X\times Y,n\in N\right).$$

Then,

$$f_{1}^{n}\times g_{1}^{n}\left(\left(x,y\right)\right)=\left(f_{n}\times g_{n}\right)\cdots \left(f_{1}\times g_{1}\right)\left(x,y\right)=\left(f_{1}^{n}\left(x\right),g_{1}^{n}\left(y\right)\right).$$

The metric $d^{\prime }$ on X×Y is given by

$$d^{\prime }\left(\left(x_{1},y_{1}\right),\left(x_{2},y_{2}\right)\right)=max\left\{d_{1}\left(x_{1},x_{2}\right),d_{2}\left(y_{1},y_{2}\right)\right\}$$

for all $\left(x_{1},y_{1}\right),\left(x_{2},y_{2}\right)\in X\times Y$.

### 2.2. Set-Valued Systems

Let K(X) be the hyperspace on *X*. That is, K(X) is the space of nonempty compact subsets of *X* with the Hausdorff metric

$$d_{H}\left(A,B\right)=max\left\{\underset{x\in A}{\sup}\underset{y\in B}{\inf}d\left(x,y\right),\underset{y\in B}{\sup}\underset{x\in A}{\inf}d\left(x,y\right)\right\}$$

for any A,B∈K(X). Clearly, $\left(K\left(X\right),d_{H}\right)$ is a compact metric space. Then the system $\left(X,f_{1,\infty }\right)$ induces a set-valued dynamical system $\left(K\left(X\right),{\bar{f}}_{1,\infty }\right)$, where ${\bar{f}}_{1,\infty }:K\left(X\right)\to K\left(X\right)$ is defined as ${\bar{f}}_{1,\infty }\left(A\right)=f_{1,\infty }\left(A\right)$ for any A∈K(X). For any finite collection $A_{1},\cdots ,A_{n}$ of nonempty subsets of *X*, let

$$\langle A_{1},\cdots ,A_{n}\rangle =\left\{A\in K\left(X\right):A\subset \overset{n}{\underset{i=1}{⋃}}A_{i},A\cap A_{i}\neq ,1\leq i\leq n\right\},$$

where the topology on K(X) given by the metric $d_{H}$ is the same as the Vietoris or finite topology, which is generated by a basis consisting of all sets of the form, $\langle U_{1},\cdots ,U_{n}\rangle$, where $\left\{U_{1},\cdots ,U_{n}\right\}$ is an arbitrary finite collection of nonempty open subsets of *X*.

### 2.3. Basic Definitions

In this section, some definitions of the shadowing properties in NDDSs are given.

**Definition** **1** ([24]). *Let P be the collection of all subsets of $Z^{+}$. A collection F⊂P is called a Furstenberg family if it is hereditary upwards, i.e., $F_{1}\subset F_{2}$ and $F_{1}\subset F$ imply $F_{2}\subset F$.*

**Definition** **2** ([10]). *For a δ>0, a sequence ${\left\{x_{i}\right\}}_{i=0}^{\infty }\subset X$ is called a δ-ergodic pseudo-orbit of $f_{1,\infty }$ if*

$$\lim_{n\to \infty }\frac{1}{n}\left|\left\{0\leq i<n:d\left(f_{i+1}\left(x_{i}\right),x_{i+1}\right)<\delta \right\}\right|=1.$$

**Definition** **3** ([10,11,12]). *An NDDS $\left(X,f_{1,\infty }\right)$ has*

*(1)*  *F-shadowing property if for any ε>0 there exists a δ>0 such that every δ-ergodic pseudo-orbit ${\left\{x_{i}\right\}}_{i=0}^{\infty }\subset X$ is F-ε-shadowed by a point z∈X, i.e., $\{i\in Z^{+}:d\left(f_{1}^{i}\left(z\right),x_{i}\right)<\varepsilon \}\in F$, where F is a Furstenberg family;*
*(2)*  *Ergodic shadowing property if for any ε>0, there exists a δ>0 such that every δ-ergodic pseudo-orbit ${\left\{x_{i}\right\}}_{i=0}^{\infty }\subset X$ is ε-ergodic shadowed by a point z∈X, i.e.,*
$$\lim_{n\to \infty }\frac{1}{n}\left|\left\{0\leq i<n:d\left(f_{1}^{i}\left(z\right),x_{i}\right)<\varepsilon \right\}\right|=1;$$
*(3)*  *$\bar{d}$ shadowing property if for any ε>0 there exists a δ>0 such that every δ-ergodic pseudo-orbit ${\left\{x_{i}\right\}}_{i=0}^{\infty }$ is ε-shadowed by a point z∈X in a way such that*
$$\underset{n\to \infty }{lim sup}\frac{1}{n}\left|\left\{0\leq i<n:d\left(f_{1}^{i}\left(z\right),x_{i}\right)<\varepsilon \right\}\right|>\frac{1}{2};$$
*(4)*  *$\underline{d}$ shadowing property if for any ε>0 there exists a δ>0 such that every δ-ergodic pseudo-orbit ${\left\{x_{i}\right\}}_{i=0}^{\infty }$ is ε-shadowed by a point z∈X in a way such that*
$$\underset{n\to \infty }{lim inf}\frac{1}{n}\left|\left\{0\leq i<n:d\left(f_{1}^{i}\left(z\right),x_{i}\right)<\varepsilon \right\}\right|>0.$$

**Definition** **4** ([25]). *Let $\left(X,d_{1}\right)$ and $\left(Y,d_{2}\right)$ be two metric spaces with non-autonomous mapping sequences $f_{1,\infty }={\left\{f_{n}\right\}}_{n=1}^{\infty }$ and $g_{1,\infty }={\left\{g_{n}\right\}}_{n=1}^{\infty }$, respectively. If there is a homeomorphism h:X→Y such that $h\circ f_{n}=g_{n}\circ h$, for all n=1,2,⋯, then $f_{1,\infty }$ and $g_{1,\infty }$ are said to be topologically conjugate.*

## 3. Main Results

### 3.1. The Retentivity of Shadowing Properties

In this section, we prove some results related to compound operation, topological conjugacy and product for NDDSs with shadowing properties.

**Theorem** **1.** *Let $\left(X,f_{1,\infty }\right)$ be an NDDS. If $\left(X,f_{1,\infty }\right)$ has the F-shadowing property (resp., ergodic shadowing property, $\bar{d}$ shadowing property, $\underline{d}$ shadowing property), then so does $\left(X,f_{1,\infty }^{\left[m\right]}\right)$.*

**Proof.**  If m=1, it is obvious. Suppose that m≥2. Let ε>0 be given. By the F-shadowing property of $f_{1,\infty }$, for any ε>0, there exists a δ>0 such that each δ-ergodic pseudo-orbit of $f_{1,\infty }$ is F-ε-shadowed by some points in *X*. Let ${\left\{y_{i}\right\}}_{i=0}^{\infty }$ be a δ-ergodic pseudo-orbit for $h_{1,\infty }=f_{1,\infty }^{\left[m\right]}$, then

$$\lim_{n\to \infty }\frac{1}{n}\left|\left\{0\leq i<n:d\left(h_{i+1}\left(y_{i}\right),y_{i+1}\right)<\delta \right\}\right|=1.$$

Since $h_{i+1}=f_{im+1}^{(i+1)m}$, then

$$\lim_{n\to \infty }\frac{1}{n}\left|\left\{0\leq i<n:d\left(f_{im+1}^{(i+1)m}\left(y_{i}\right),y_{i+1}\right)<\delta \right\}\right|=1.$$

For 0≤j<m and i≥0, put $x_{im+j}=f_{im+1}^{im+j}\left(y_{i}\right)$. One can claim that ${\left\{x_{i}\right\}}_{i=0}^{\infty }$ is a δ-ergodic pseudo-orbit for $f_{1,\infty }$. So

$$\lim_{n\to \infty }\frac{1}{n}\left|\left\{0\leq i<n:d\left(f_{im+j+1}\left(x_{im+j}\right),x_{im+j+1}\right)<\delta \right\}\right|=1$$

for 0≤j<m. Choose any i≥0. For any j:0≤j≤m−2,

$$f_{im+j+1}\left(x_{im+j}\right)=f_{im+j+1}\left(f_{im+1}^{im+j}\left(y_{i}\right)\right)=f_{im+1}^{im+j+1}\left(y_{i}\right)=x_{im+j+1}.$$

Therefore, $d(f_{im+j+1}\left(x_{im+j}\right),x_{im+j+1})=0<\delta$ for all j:0≤j≤m−2. Now for j=m−1, for every k∈N, set $x_{mk}=y_{k}$, then

$$\lim_{n\to \infty }\frac{1}{n}\left|\left\{0\leq i<n:d\left(f_{im+m}\left(x_{im+m-1}\right),x_{im+m}\right)<\delta \right\}\right|=1,$$

where

$$\begin{array}{cc} d(f_{im+m}\left(x_{im+m-1}\right),x_{im+m}) & =d(f_{im+m}\left(f_{im+1}^{im+m-1}\left(y_{i}\right)\right),x_{(i+1)m})\\ & =d(f_{im+1}^{(i+1)m}\left(y_{i}\right),y_{i+1})\\ & =d(h_{i+1}\left(y_{i}\right),y_{i+1})\\ & <\delta . \end{array}$$

Hence ${\left\{x_{i}\right\}}_{i=0}^{\infty }$ is a δ-ergodic pseudo-orbit for $f_{1,\infty }$. So, by the F-shadowing property of $\left(X,f_{1,\infty }\right)$, there is a point z∈X such that ${\left\{x_{i}\right\}}_{i=0}^{\infty }$ is F-ε-shadowed by *z*, that is,

$$\{i\in Z^{+}:d\left(f_{1}^{i}\left(z\right),x_{i}\right)<\varepsilon \}\in F.$$

In particular, taking the value of index *i* being mi, one has

$$\begin{array}{cc} \left\{i\in Z^{+}:d\left(f_{1}^{mi}\left(z\right),x_{mi}\right)<\varepsilon \right\} & =\{i\in Z^{+}:d\left(f_{1}^{mi}\left(z\right),y_{i}\right)<\varepsilon \}\\ & =\{i\in Z^{+}:d\left(h_{1}^{i}\left(z\right),y_{i}\right)<\varepsilon \}\\ & \in F. \end{array}$$

So, *z* is a point of *X* which is F-ε-shadowing the δ-ergodic pseudo-orbit ${\left\{y_{i}\right\}}_{i=0}^{\infty }$ of $f_{1,\infty }^{\left[m\right]}$. Thus, $\left(X,f_{1,\infty }^{\left[m\right]}\right)$ has a F-shadowing property.

Similarly, one can prove the results about the ergodic shadowing property, $\bar{d}$ shadowing property, and $\underline{d}$ shadowing property. □

**Theorem** **2.** *Let $\left(X,d_{1}\right)$ and $\left(Y,d_{2}\right)$ be metric spaces with non-autonomous mapping sequences $f_{1,\infty }$ and $g_{1,\infty }$ defined on them, respectively. If $f_{1,\infty }$ is topologically conjugate to $g_{1,\infty }$, then $\left(X,f_{1,\infty }\right)$ has an ergodic shadowing property (resp., $\bar{d}$ shadowing property, $\underline{d}$ shadowing property, and F-shadowing property) if, and only if, $\left(Y,g_{1,\infty }\right)$ also does.*

**Proof.**  Let $\varepsilon_{2}>0$ be given. Since $f_{1,\infty }$ is topologically conjugate to $g_{1,\infty }$, there exists a homeomorphism h:X→Y such that $h\circ f_{n}=g_{n}\circ h$ or $f_{n}\circ h^{-1}=h^{-1}\circ g_{n}$ for all n≥0. By the uniform continuity of *h*, for every $\varepsilon_{2}>0$, $d_{1}\left(x,y\right)<\varepsilon_{1}$ for a $\varepsilon_{1}>0$ implies $d_{2}\left(h\left(x\right),h\left(y\right)\right)<\varepsilon_{2}$. Since $f_{1,\infty }$ has an ergodic shadowing property, then for the above $\varepsilon_{1}>0$, there exists a $\delta_{1}>0$ such that, $\delta_{1}$-ergodic pseudo-orbit ${\left\{x_{i}\right\}}_{i=0}^{\infty }$ of $f_{1,\infty }$ can be $\varepsilon_{1}$-ergodic shadowed by some points in *X*. By the uniform continuity of $h^{-1}$, for $\delta_{1}>0$, there is a $\delta_{2}>0$ such that $d_{2}\left(x,y\right)<\delta_{2}$ implies $d_{1}\left(h^{-1}\left(x\right),h^{-1}\left(y\right)\right)<\delta_{1}$. The following will prove that each $\delta_{2}$-ergodic pseudo-orbit of $g_{1,\infty }$ can be $\varepsilon_{2}$-ergodic shadowed by some points in *Y*.

Suppose that ${\left\{y_{i}\right\}}_{i=0}^{\infty }$ is a $\delta_{2}$-ergodic pseudo-orbit of $g_{1,\infty }$, and put $x_{i}=h^{-1}\left(y_{i}\right)$ for all i∈N. Since

$$\lim_{n\to \infty }\frac{1}{n}\left|\left\{0\leq i<n:d_{2}\left(g_{i+1}\left(y_{i}\right),y_{i+1}\right)<\delta_{2}\right\}\right|=1,$$

then

$$\lim_{n\to \infty }\frac{1}{n}\left|\left\{0\leq i<n:d_{1}\left(h^{-1}\left(g_{i+1}\left(y_{i}\right)\right),h^{-1}\left(y_{i+1}\right)\right)<\delta_{1}\right\}\right|=1,$$

so

$$\begin{array}{cc} & \lim_{n\to \infty }\frac{1}{n}\left|\left\{0\leq i<n:d_{1}\left(h^{-1}\left(g_{i+1}\left(y_{i}\right)\right),h^{-1}\left(y_{i+1}\right)\right)<\delta_{1}\right\}\right|\\ & =\lim_{n\to \infty }\frac{1}{n}\left|\left\{0\leq i<n:d_{1}\left(f_{i+1}\left(h^{-1}\left(y_{i}\right)\right),h^{-1}\left(y_{i+1}\right)\right)<\delta_{1}\right\}\right|\\ & =\lim_{n\to \infty }\frac{1}{n}\left|\left\{0\leq i<n:d_{1}\left(f_{i+1}\left(x_{i}\right),x_{i+1}\right)<\delta_{1}\right\}\right|\\ & =1. \end{array}$$

Thus ${\left\{x_{i}\right\}}_{i=0}^{\infty }$ is a $\delta_{1}$-ergodic pseudo-orbit of $f_{1,\infty }$ and there exists a z∈X such that

$$\lim_{n\to \infty }\frac{1}{n}\left|\left\{0\leq i<n:d_{1}\left(f_{1}^{i}\left(z\right),x_{i}\right)<\varepsilon_{1}\right\}\right|=1.$$

Let $F_{i}=f_{1}^{i}=f_{i}\circ \cdots \circ f_{1},G_{i}=g_{1}^{i}=g_{i}\circ \cdots \circ g_{1}$, then

$$\begin{array}{cc} & \lim_{n\to \infty }\frac{1}{n}\left|\left\{0\leq i<n:d_{1}\left(F_{i}\left(z\right),x_{i}\right)<\varepsilon_{1}\right\}\right|\\ & =\lim_{n\to \infty }\frac{1}{n}\left|\left\{0\leq i<n:d_{2}\left(h\left(F_{i}\left(z\right)\right),h\left(x_{i}\right)\right)<\varepsilon_{2}\right\}\right|\\ & =\lim_{n\to \infty }\frac{1}{n}\left|\left\{0\leq i<n:d_{2}\left(G_{i}\left(h\left(z\right)\right),y_{i}\right)<\varepsilon_{2}\right\}\right|\\ & =1. \end{array}$$

So h(z) is the point in $\left(Y,g_{1,\infty }\right)$ that ${\left\{y_{i}\right\}}_{i=0}^{\infty }$ is $\varepsilon_{2}$-ergodic shadowed. Therefore, $\left(Y,g_{1,\infty }\right)$ has an ergodic shadowing property.

On the other hand, let $\left(Y,g_{1,\infty }\right)$ has an ergodic shadowing property, one can prove that $\left(X,f_{1,\infty }\right)$ has an ergodic shadowing property.

The proofs of the $\bar{d}$ shadowing property, $\underline{d}$ shadowing property and F-shadowing property are similar to that given above. □

**Theorem** **3.** *Let $\left(X,d_{1}\right)$ and $\left(Y,d_{2}\right)$ be metric spaces with non-autonomous mapping sequences $f_{1,\infty }$ and $g_{1,\infty }$ defined on them, respectively. Then*

*(1)*  *$\left(X,f_{1,\infty }\right)$ and $\left(Y,g_{1,\infty }\right)$ have a $\bar{d}$ shadowing property if, and only if, the product system $\left(X\times Y,f_{1,\infty }\times g_{1,\infty }\right)$ also does;*
*(2)*  *$\left(X,f_{1,\infty }\right)$ and $\left(Y,g_{1,\infty }\right)$ have a $\underline{d}$ shadowing property if, and only if, the product system $\left(X\times Y,f_{1,\infty }\times g_{1,\infty }\right)$ also does;*
*(3)*  *$\left(X,f_{1,\infty }\right)$ and $\left(Y,g_{1,\infty }\right)$ have F-shadowing property if, and only if, the product system $\left(X\times Y,f_{1,\infty }\times g_{1,\infty }\right)$ also does;*
*(4)*  *$\left(X,f_{1,\infty }\right)$ and $\left(Y,g_{1,\infty }\right)$ have an ergodic shadowing property if, and only if, the product system $\left(X\times Y,f_{1,\infty }\times g_{1,\infty }\right)$ also does.*

**Proof.**  (1) (Necessity) Let ε>0, then there exists a $\delta_{1}>0$ such that every $\delta_{1}$-ergodic pseudo-orbit ${\left\{x_{i}\right\}}_{i=0}^{\infty }$ of $\left(X,f_{1,\infty }\right)$ can be ε-shadowed by some points of $\left(X,f_{1,\infty }\right)$, and there exists a $\delta_{2}>0$ such that every $\delta_{2}$-ergodic pseudo-orbit ${\left\{y_{i}\right\}}_{i=0}^{\infty }$ of $\left(Y,g_{1,\infty }\right)$ can be ε-shadowed by some points of $\left(Y,g_{1,\infty }\right)$. Choose $\delta =max\{\delta_{1},\delta_{2}\}$. Then

$$\lim_{n\to \infty }\frac{1}{n}\left|\left\{0\leq i<n:d_{1}\left(f_{i+1}\left(x_{i}\right),x_{i+1}\right)<\delta \right\}\right|=1,$$

$$\lim_{n\to \infty }\frac{1}{n}\left|\left\{0\leq i<n:d_{2}\left(g_{i+1}\left(y_{i}\right),y_{i+1}\right)<\delta \right\}\right|=1.$$

So

$$\begin{array}{cc} & \lim_{n\to \infty }\frac{1}{n}\left|\left\{0\leq i<n:d^{\prime }\left(\left(f_{i+1}\times g_{i+1}\right)\left(x_{i},y_{i}\right),\left(x_{i+1},y_{i+1}\right)\right)<\delta \right\}\right|\\ & =\lim_{n\to \infty }\frac{1}{n}|\{0\leq i<n:max\left\{d_{1}\left(f_{i+1}\left(x_{i}\right),x_{i+1}\right),d_{2}\left(g_{i+1}\left(y_{i}\right),y_{i+1}\right)\right\}<\delta |\\ & =1. \end{array}$$

Therefore, ${\left\{\left(x_{i},y_{i}\right)\right\}}_{i=0}^{\infty }$ is a δ-ergodic pseudo-orbit for $\left(X\times Y,f_{1,\infty }\times g_{1,\infty }\right)$.

Assume that the NDDSs $\left(X,f_{1,\infty }\right)$ and $\left(Y,g_{1,\infty }\right)$ both have the $\bar{d}$ shadowing property. Then there exist a∈X and b∈Y such that

$$\underset{n\to \infty }{lim sup}\frac{1}{n}\left|\left\{0\leq i<n:d_{1}\left(f_{1}^{i}\left(a\right),x_{i}\right)<\varepsilon \right\}\right|>\frac{1}{2},$$

$$\underset{n\to \infty }{lim sup}\frac{1}{n}\left|\left\{0\leq i<n:d_{2}\left(g_{1}^{i}\left(b\right),y_{i}\right)<\varepsilon \right\}\right|>\frac{1}{2}.$$

Since

$$d^{\prime }\left(\left(f_{1}^{i}\times g_{1}^{i}\right)\left(a,b\right),\left(x_{i},y_{i}\right)\right)=max\left\{d_{1}\left(f_{1}^{i}\left(a\right),x_{i}\right),d_{2}\left(g_{1}^{i}\left(b\right),y_{i}\right)\right\}<\varepsilon ,$$

then

$$\underset{n\to \infty }{lim sup}\frac{1}{n}\left|\left\{0\leq i<n:d^{\prime }\left(\left(f_{1}^{i}\times g_{1}^{i}\right)\left(a,b\right),\left(x_{i},y_{i}\right)\right)<\varepsilon \right\}\right|>\frac{1}{2}.$$

Thus, the δ-ergodic pseudo-orbit ${\left\{\left(x_{i},y_{i}\right)\right\}}_{i=0}^{\infty }$ is ε-shadowed by a point (a,b) in X×Y. That is to say, $\left(X\times Y,f_{1,\infty }\times g_{1,\infty }\right)$ has a $\bar{d}$ shadowing property.

(Sufficiency) Suppose that $\left(X\times Y,f_{1,\infty }\times g_{1,\infty }\right)$ has a $\bar{d}$ shadowing property, then for any ε>0, there exists a δ>0 such that δ-ergodic pseudo-orbit ${\left\{\left(x_{i},y_{i}\right)\right\}}_{i=0}^{\infty }$ of $f_{1,\infty }\times g_{1,\infty }$ can be ε-shadowed by a point (a,b) in X×Y. Then

$$\underset{n\to \infty }{lim sup}\frac{1}{n}\left|\left\{0\leq i<n:d^{\prime }\left(\left(f_{1}^{i}\times g_{1}^{i}\right)\left(a,b\right),\left(x_{i},y_{i}\right)\right)<\varepsilon \right\}\right|>\frac{1}{2}.$$

Since

$$d^{\prime }\left(\left(f_{1}^{i}\times g_{1}^{i}\right)\left(a,b\right),\left(x_{i},y_{i}\right)\right)=max\left\{d_{1}\left(f_{1}^{i}\left(a\right),x_{i}\right),d_{2}\left(g_{1}^{i}\left(b\right),y_{i}\right)\right\},$$

then

$$d_{1}\left(f_{1}^{i}\left(a\right),x_{i}\right)\leq d^{\prime }\left(\left(f_{1}^{i}\times g_{1}^{i}\right)\left(a,b\right),\left(x_{i},y_{i}\right)\right)<\varepsilon ,$$

$$d_{2}\left(g_{1}^{i}\left(b\right),y_{i}\right)\leq d^{\prime }\left(\left(f_{1}^{i}\times g_{1}^{i}\right)\left(a,b\right),\left(x_{i},y_{i}\right)\right)<\varepsilon .$$

Thus

$$\underset{n\to \infty }{lim sup}\frac{1}{n}\left|\left\{0\leq i<n:d_{1}\left(f_{1}^{i}\left(a\right),x_{i}\right)<\varepsilon \right\}\right|>\frac{1}{2},$$

$$\underset{n\to \infty }{lim sup}\frac{1}{n}\left|\left\{0\leq i<n:d_{2}\left(g_{1}^{i}\left(b\right),y_{i}\right)<\varepsilon \right\}\right|>\frac{1}{2}.$$

Because ${\left\{\left(x_{i},y_{i}\right)\right\}}_{i=0}^{\infty }$ is a δ-ergodic pseudo-orbit of $\left(X\times Y,f_{1,\infty }\times g_{1,\infty }\right)$, it is easy to obtain that ${\left\{x_{i}\right\}}_{i=0}^{\infty }$, ${\left\{y_{i}\right\}}_{i=0}^{\infty }$ are δ-ergodic pseudo-orbit of $f_{1,\infty }$ and $g_{1,\infty }$, respectively. Therefore, a δ-ergodic pseudo-orbit ${\left\{x_{i}\right\}}_{i=0}^{\infty }$ is ε-shadowed by a point *a* in *X*, and a δ-ergodic pseudo-orbit ${\left\{y_{i}\right\}}_{i=0}^{\infty }$ is ε-shadowed by a point *b* in *Y*. Hence $\left(X,f_{1,\infty }\right)$ and $\left(Y,g_{1,\infty }\right)$ have a $\bar{d}$ shadowing property.

(2) The proof is similar to (1).

(3) (Necessity) Let ε>0, there exist $\delta_{1}>0$ and $\delta_{2}>0$ such that every $\delta_{1}$-ergodic pseudo-orbit of $\left(X,f_{1,\infty }\right)$ and every $\delta_{2}$-ergodic pseudo-orbit of $\left(Y,g_{1,\infty }\right)$ can be F-ε-shadowed by some point of $\left(X,f_{1,\infty }\right)$ and $\left(Y,g_{1,\infty }\right)$, respectively. Choose $\delta =max\{\delta_{1},\delta_{2}\}$ and let ${\left\{\left(x_{i},y_{i}\right)\right\}}_{i=0}^{\infty }$ be a δ-ergodic pseudo-orbit for $\left(X\times Y,f_{1,\infty }\times g_{1,\infty }\right)$.

Assume that the NDDSs $\left(X,f_{1,\infty }\right)$ and $\left(Y,g_{1,\infty }\right)$ both have the F-shadowing property. Then, there exist a∈X and b∈Y such that

$$\{i\in Z^{+}:d_{1}\left(f_{1}^{i}\left(a\right),x_{i}\right)<\varepsilon \}\in F,$$

$$\{i\in Z^{+}:d_{2}\left(g_{1}^{i}\left(b\right),y_{i}\right)<\varepsilon \}\in F.$$

Since

$$d^{\prime }\left(\left(f_{1}^{i}\times g_{1}^{i}\right)\left(a,b\right),\left(x_{i},y_{i}\right)\right)=max\left\{d_{1}\left(f_{1}^{i}\left(a\right),x_{i}\right),d_{2}\left(g_{1}^{i}\left(b\right),y_{i}\right)\right\},$$

then

$$\{i\in Z^{+}:d^{\prime }\left(\left(f_{1}^{i}\times g_{1}^{i}\right)\left(a,b\right),\left(x_{i},y_{i}\right)\right)<\varepsilon \}\in F.$$

Thus, the δ-ergodic pseudo-orbit ${\left\{\left(x_{i},y_{i}\right)\right\}}_{i=0}^{\infty }$ is F-ε-shadowed by a point (a,b) in X×Y, i.e., $\left(X\times Y,f_{1,\infty }\times g_{1,\infty }\right)$ has a F-shadowing property.

(Sufficiency) Suppose that $\left(X\times Y,f_{1,\infty }\times g_{1,\infty }\right)$ has a F-shadowing property, then for any ε>0, there exists a δ>0 such that δ-ergodic pseudo-orbit ${\left\{\left(x_{i},y_{i}\right)\right\}}_{i=0}^{\infty }$ of $f_{1,\infty }\times g_{1,\infty }$ can be F-ε-shadowed by a point (a,b) in X×Y. Then

$$\{i\in Z^{+}:d^{\prime }\left(\left(f_{1}^{i}\times g_{1}^{i}\right)\left(a,b\right),\left(x_{i},y_{i}\right)\right)<\varepsilon \}\in F.$$

For any integer $i\in \{i\in Z^{+}:d^{\prime }\left(\left(f_{1}^{i}\times g_{1}^{i}\right)\left(a,b\right),\left(x_{i},y_{i}\right)\right)<\varepsilon \}$, one can obtain

$$d_{1}\left(f_{1}^{i}\left(a\right),x_{i}\right)\leq max\{d_{1}\left(f_{1}^{i}\left(a\right),x_{i}\right),d_{2}\left(g_{1}^{i}\left(b\right),y_{i}\right)=d^{\prime }\left(\left(f_{1}^{i}\times g_{1}^{i}\right)\left(a,b\right),\left(x_{i},y_{i}\right)\right)<\varepsilon ,$$

$$d_{2}\left(g_{1}^{i}\left(b\right),y_{i}\right)\leq max\{d_{1}\left(f_{1}^{i}\left(a\right),x_{i}\right),d_{2}\left(g_{1}^{i}\left(b\right),y_{i}\right)=d^{\prime }\left(\left(f_{1}^{i}\times g_{1}^{i}\right)\left(a,b\right),\left(x_{i},y_{i}\right)\right)<\varepsilon .$$

Thus,

$$\{i\in Z^{+}:d_{1}\left(f_{1}^{i}\left(a\right),x_{i}\right)<\varepsilon \}\in F,$$

$$\{i\in Z^{+}:d_{2}\left(g_{1}^{i}\left(b\right),y_{i}\right)<\varepsilon \}\in F.$$

Therefore, the δ-ergodic pseudo-orbit ${\left\{x_{i}\right\}}_{i=0}^{\infty }$ of $f_{1,\infty }$ is F-ε-shadowed by a point *a* in *X*, the δ-ergodic pseudo-orbit ${\left\{y_{i}\right\}}_{i=0}^{\infty }$ of $g_{1,\infty }$ is F-ε-shadowed by a point *b* in *Y*. Hence $\left(X,f_{1,\infty }\right)$ and $\left(Y,g_{1,\infty }\right)$ have the F-shadowing property.

(4) (Necessity) Let ε>0, then there exist $\delta_{1}>0$ and $\delta_{2}>0$ such that every $\delta_{1}$-ergodic pseudo-orbit of $\left(X,f_{1,\infty }\right)$ and every $\delta_{2}$-ergodic pseudo-orbit of $\left(Y,g_{1,\infty }\right)$ can be ε-ergodic shadowed by some points of $\left(X,f_{1,\infty }\right)$ and $\left(Y,g_{1,\infty }\right)$, respectively. Choose $\delta =max\{\delta_{1},\delta_{2}\}$ and let ${\left\{\left(x_{i},y_{i}\right)\right\}}_{i=0}^{\infty }$ be a δ-ergodic pseudo-orbit for $\left(X\times Y,f_{1,\infty }\times g_{1,\infty }\right)$.

Assume that the NDDSs $\left(X,f_{1,\infty }\right)$ and $\left(Y,g_{1,\infty }\right)$ both have the ergodic shadowing property. Then, there exist a∈X and b∈Y such that

$$\lim_{n\to \infty }\frac{1}{n}\left|\left\{0\leq i<n:d_{1}\left(f_{1}^{i}\left(a\right),x_{i}\right)<\varepsilon \right\}\right|=1,$$

$$\lim_{n\to \infty }\frac{1}{n}\left|\left\{0\leq i<n:d_{2}\left(g_{1}^{i}\left(b\right),y_{i}\right)<\varepsilon \right\}\right|=1.$$

Similar to (3), one can obtain

$$\lim_{n\to \infty }\frac{1}{n}\left|\left\{0\leq i<n:d^{\prime }\left(\left(f_{1}^{i}\times g_{1}^{i}\right)\left(a,b\right),\left(x_{i},y_{i}\right)\right)<\varepsilon \right\}\right|=1.$$

So, the δ-ergodic pseudo-orbit ${\left\{\left(x_{i},y_{i}\right)\right\}}_{i=0}^{\infty }$ is ε-ergodic shadowed by a point (a,b) in X×Y. Thus, $\left(X\times Y,f_{1,\infty }\times g_{1,\infty }\right)$ has an ergodic shadowing property.

(Sufficiency) Suppose that $\left(X\times Y,f_{1,\infty }\times g_{1,\infty }\right)$ has an ergodic shadowing property, then for any ε>0, there exists a δ>0 such that δ-ergodic pseudo-orbit ${\left\{\left(x_{i},y_{i}\right)\right\}}_{i=0}^{\infty }$ of $f_{1,\infty }\times g_{1,\infty }$ can be ε-ergodic shadowed by a point (a,b) in X×Y. Then

$$\lim_{n\to \infty }\frac{1}{n}\left|\left\{0\leq i<n:d^{\prime }\left(\left(f_{1}^{i}\times g_{1}^{i}\right)\left(a,b\right),\left(x_{i},y_{i}\right)\right)<\varepsilon \right\}\right|=1.$$

So

$$d_{1}\left(f_{1}^{i}\left(a\right),x_{i}\right)\leq max\{d_{1}\left(f_{1}^{i}\left(a\right),x_{i}\right),d_{2}\left(g_{1}^{i}\left(b\right),y_{i}\right)=d^{\prime }\left(\left(f_{1}^{i}\times g_{1}^{i}\right)\left(a,b\right),\left(x_{i},y_{i}\right)\right)<\varepsilon ,$$

$$d_{2}\left(g_{1}^{i}\left(b\right),y_{i}\right)\leq max\{d_{1}\left(f_{1}^{i}\left(a\right),x_{i}\right),d_{2}\left(g_{1}^{i}\left(b\right),y_{i}\right)=d^{\prime }\left(\left(f_{1}^{i}\times g_{1}^{i}\right)\left(a,b\right),\left(x_{i},y_{i}\right)\right)<\varepsilon .$$

Thus

$$\lim_{n\to \infty }\frac{1}{n}\left|\left\{0\leq i<n:d_{1}\left(f_{1}^{i}\left(a\right),x_{i}\right)<\varepsilon \right\}\right|=1,$$

$$\lim_{n\to \infty }\frac{1}{n}\left|\left\{0\leq i<n:d_{2}\left(g_{1}^{i}\left(b\right),y_{i}\right)<\varepsilon \right\}\right|=1.$$

Therefore, the δ-ergodic pseudo-orbit ${\left\{x_{i}\right\}}_{i=0}^{\infty }$ of $f_{1,\infty }$ is ε-ergodic shadowed by a point *a* in *X*, the δ-ergodic pseudo-orbit ${\left\{y_{i}\right\}}_{i=0}^{\infty }$ of $g_{1,\infty }$ is ε-ergodic shadowed by a point *b* in *Y*. Hence $\left(X,f_{1,\infty }\right)$ and $\left(Y,g_{1,\infty }\right)$ have an ergodic shadowing property. □

### 3.2. The Relationship of Shadowing Properties between $f_{1,\infty }$ and ${\bar{f}}_{1,\infty }$

Now, the relationship of the above four kinds of shadowing properties between NDDSs and the set-valued systems are discussed.

**Theorem** **4.** *Let $\left(K\left(X\right),{\bar{f}}_{1,\infty }\right)$ be a set-valued dynamical system induced by $\left(X,f_{1,\infty }\right)$.*

*(1)*  *If $\left(K\left(X\right),{\bar{f}}_{1,\infty }\right)$ has a F-shadowing property, then $\left(X,f_{1,\infty }\right)$ has a F-shadowing property;*
*(2)*  *If $\left(K\left(X\right),{\bar{f}}_{1,\infty }\right)$ has an ergodic shadowing property, then $\left(X,f_{1,\infty }\right)$ has an ergodic shadowing property;*
*(3)*  *If $\left(K\left(X\right),{\bar{f}}_{1,\infty }\right)$ has a $\bar{d}$ shadowing property, then $\left(X,f_{1,\infty }\right)$ has a $\bar{d}$ shadowing property;*
*(4)*  *If $\left(K\left(X\right),{\bar{f}}_{1,\infty }\right)$ has a $\underline{d}$ shadowing property, then $\left(X,f_{1,\infty }\right)$ has a $\underline{d}$ shadowing property.*

**Proof.**  (1) Let ε>0, then there exists a δ>0 such that every δ-ergodic pseudo-orbit of ${\bar{f}}_{1,\infty }$ is F-ε-shadowed by some elements of K(X). Let $\left\{x_{i}:i\in N\right\}$ be a δ-ergodic pseudo-orbit of $f_{1,\infty }$, then $\left\{\left\{x_{i}\right\}:i\in N\right\}$ is a δ-ergodic pseudo-orbit of ${\bar{f}}_{1,\infty }$. Since

$$\lim_{n\to \infty }\frac{1}{n}\left|\left\{0\leq i<n:d_{H}\left({\bar{f}}_{i+1}\left(\left\{x_{i}\right\}\right),\left\{x_{i+1}\right\}\right)<\delta \right\}\right|=1,$$

where

$$d_{H}\left({\bar{f}}_{i+1}\left(\left\{x_{i}\right\}\right),\left\{x_{i+1}\right\}\right)=d\left(f_{i+1}\left(x_{i}\right),x_{i+1}\right),$$

then

$$\lim_{n\to \infty }\frac{1}{n}\left|\left\{0\leq i<n:d\left(f_{i+1}\left(x_{i}\right),x_{i+1}\right)<\delta \right\}\right|=1.$$

Then $\left\{x_{i}:i\in N\right\}$ is a δ-ergodic pseudo-orbit of $f_{1,\infty }$. So we can find an element A∈K(X) such that $\{i\in Z^{+}:d_{H}\left({\bar{f}}_{1}^{i}\left(A\right),\left\{x_{i}\right\}\right)<\varepsilon \}\in F$, where

$$d_{H}\left({\bar{f}}_{1}^{i}\left(A\right),\left\{x_{i}\right\}\right)=\underset{y\in A}{\sup}d\left(f_{1}^{i}\left(y\right),x_{i}\right)<\varepsilon$$

for all $i\in Z^{+}$. So, $d(f_{1}^{i}\left(y\right),x_{i})<\varepsilon$ for any y∈A and all $i\in Z^{+}$. Then $\{i\in Z^{+}:d\left(f_{1}^{i}\left(y\right),x_{i}\right)<\varepsilon \}\in F$. Hence, for given ε>0, there is a δ>0 such that every δ-ergodic pseudo-orbit $\left\{x_{i}:i\in N\right\}$ of $f_{1,\infty }$ is F-ε-shadowed by some y∈X. This implies that $f_{1,\infty }$ has a F-shadowing property.

(2) Let ε>0, then there exists a δ>0 such that every δ-ergodic pseudo-orbit of ${\bar{f}}_{1,\infty }$ is ε-ergodic shadowed by some element of K(X). Let $\left\{x_{i}:i\in N\right\}$ be a δ-ergodic pseudo-orbit of $f_{1,\infty }$ and $\left\{\left\{x_{i}\right\}:i\in N\right\}$ is a δ-ergodic pseudo-orbit of ${\bar{f}}_{1,\infty }$. One can find an element A∈K(X) such that

$$\lim_{n\to \infty }\frac{1}{n}\left|\left\{0\leq i<n:d_{H}\left({\bar{f}}_{1}^{i}\left(A\right),\left\{x_{i}\right\}\right)<\varepsilon \right\}\right|=1,$$

where

$$d_{H}\left({\bar{f}}_{1}^{i}\left(A\right),\left\{x_{i}\right\}\right)=\underset{y\in A}{\sup}d\left(f_{1}^{i}\left(y\right),x_{i}\right)<\varepsilon$$

for all i∈N. So, $d(f_{1}^{i}\left(y\right),x_{i})<\varepsilon$ for any y∈A and all i∈N. Then

$$\lim_{n\to \infty }\frac{1}{n}\left|\left\{0\leq i<n:d\left(f_{1}^{i}\left(y\right),x_{i}\right)<\varepsilon \right\}\right|=1.$$

Hence, for given ε>0, there is a δ>0 such that every δ-ergodic pseudo-orbit $\left\{x_{i}:i\in N\right\}$ of $f_{1,\infty }$ is ε-ergodic shadowed by some y∈X. This implies that $f_{1,\infty }$ has an ergodic shadowing property.

(3) Let ε>0, then there exists a δ>0 such that every δ-ergodic pseudo-orbit of ${\bar{f}}_{1,\infty }$ is ε-shadowed by some elements of K(X). Let $\left\{x_{i}:i\in N\right\}$ be a δ-ergodic pseudo-orbit of $f_{1,\infty }$ and $\left\{\left\{x_{i}\right\}:i\in N\right\}$ be a δ-ergodic pseudo-orbit of ${\bar{f}}_{1,\infty }$. One can find an element A∈K(X) such that

$$\underset{n\to \infty }{lim sup}\frac{1}{n}\left|\left\{0\leq i<n:d_{H}\left({\bar{f}}_{1}^{i}\left(A\right),\left\{x_{i}\right\}\right)<\varepsilon \right\}\right|>\frac{1}{2},$$

where

$$d_{H}\left({\bar{f}}_{1}^{i}\left(A\right),\left\{x_{i}\right\}\right)=\underset{y\in A}{\sup}d\left(f_{1}^{i}\left(y\right),x_{i}\right)<\varepsilon$$

for all i∈N. So any y∈A will satisfy $d(f_{1}^{i}\left(y\right),x_{i})<\varepsilon$ for all i∈N. Then

$$\underset{n\to \infty }{lim sup}\frac{1}{n}\left|\left\{0\leq i<n:d\left(f_{1}^{i}\left(y\right),x_{i}\right)<\varepsilon \right\}\right|>\frac{1}{2}.$$

Hence, for given ε>0, there is a δ>0 such that every δ-ergodic pseudo-orbit $\left\{x_{i}:i\in N\right\}$ of $f_{1,\infty }$ is ε-shadowed by some y∈X. This implies that $f_{1,\infty }$ has a $\bar{d}$ shadowing property.

(4) The proof is similar to (3). □

## 4. Conclusions

In this paper, under the cases of compound, topological conjugate, or product, the retentivity of four kinds of shadowing properties are obtained. Moreover, it is proved that the shadowing properties of ${\bar{f}}_{1,\infty }$ imply the shadowing properties of $f_{1,\infty }$. However, this paper does not obtain the inverse as being true. Is the reverse of Theorem 4 true? Moreover, are other kinds of shadowing properties consistent under topological conjugation (resp. compound and product)? There remain many problems to study in the future.

## Sample Availability

Samples of the compounds are available from the corresponding author.
